#  Integrated routine workflow using next-generation sequencing and a fully-automated platform for the detection of *KRAS, NRAS* and *BRAF* mutations in formalin-fixed paraffin embedded samples with poor DNA quality in patients with colorectal carcinoma

**DOI:** 10.1371/journal.pone.0212801

**Published:** 2019-02-27

**Authors:** Claire Franczak, Ludovic Dubouis, Pauline Gilson, Marie Husson, Marie Rouyer, Jessica Demange, Agnès Leroux, Jean-Louis Merlin, Alexandre Harlé

**Affiliations:** 1 Université de Lorraine, CNRS UMR 7039 CRAN, Institut de Cancérologie de Lorraine, Service de Biopathologie, Nancy, France; 2 Institut de Cancérologie de Lorraine, Service de Biopathologie, Nancy, France; Universita degli Studi di Napoli Federico II, ITALY

## Abstract

**Background:**

*KRAS* and *NRAS* mutations are identified resistance mutations to anti-epidermal growth factor receptor monoclonal antibodies in patients with metastatic colorectal cancer. *BRAF* status is also routinely assessed for its poor prognosis value. In our institute, next-generation sequencing (NGS) is routinely used for gene-panel mutations detection including *KRAS*, *NRAS* and *BRAF*, but DNA quality is sometimes not sufficient for sequencing. In our routine practice, Idylla platform is used for the analysis of samples that don’t reach sufficient quality criteria for NGS assay.

**Methods:**

In this study, data from mCRC samples analyzed from May 2017 to 2018 were retrospectively collected. All samples with a poor DNA quality for sequencing have been assessed using Idylla platform. First, *KRAS* Idylla assay cartridge has been used for the determination of *KRAS* mutational status. All *KRAS* wild-type samples have then been analyzed using *NRAS-BRAF* assay. Among 669 samples, 67 samples failed the DNA quality control and have been assessed on Idylla *KRAS* mutation test.

**Results:**

Among 67 samples, 50 (75%) samples had a valid result with Idylla *KRAS* mutation test including 22 carrying a *KRAS* mutation. For 28 samples, *NRAS* and *BRAF* mutational statuses have been assessed using Idylla *NRAS-BRAF* mutation test. Among 28 samples, 27 (96%) had a valid result including 2 samples bearing a *NRAS* mutation and 3 samples bearing a *BRAF* mutation.

**Conclusions:**

Our study shows that an integrated workflow using NGS and Idylla platform allows the determination of *KRAS*, *NRAS* and *BRAF* mutational statuses of 651/669 (97.3%) samples and retrieve 49/67 (73.1%)samples that don’t reach DNA quality requirements for NGS.

## Introduction

Colorectal cancer (CRC) is the third most common cancer in men and the second in women worldwide [1]. Despite current early detection strategies for CRC, 20% of CRC are diagnosed at a metastatic stage [2,3].

Use of anti-epidermal growth factor receptor monoclonal antibodies (anti-EGFR mAbs) has improved overall outcome of patients with metastatic CRC (mCRC). Tumor *KRAS* and *NRAS* mutational statuses are a prerequisite for the prescription of anti-EGFR mAbs. Wild-type *KRAS* and *NRAS* statuses predict early response to anti-EGFR mAbs treatment whereas *KRAS* and *NRAS* hotspot mutations are associated with clinical resistance to anti-EGFR mAbs [4–6].

In mCRC context, *BRAF* mutational status is also commonly assessed because the presence of a V600E mutation is recognized as a poor prognosis factor [7]. According to these data, in mCRC context, research of *KRAS*, *NRAS* and *BRAF* somatic mutations has become a routine practice. Several sequencing or PCR-based assays are available for molecular sample characterization. According to guidelines, *RAS* analysis should include at least *KRAS* exons 2, 3 and 4 (codons 12, 13, 59, 61, 117 and 146) and *NRAS* exons 2, 3 and 4 (codons 12, 13, 59, 61 and 117). Turnaround time for *RAS* testing should be less than 7 working days from the time of receipt of the specimen by the testing laboratory to the time of issuing of the final report, for more than 90% of specimens. For prognostic assessment, tumor *BRAF* mutational status should be assessed alongside the assessment of tumor *RAS* mutational status [4].

Still according to these guidelines, next-generation sequencing (NGS) has been chosen for routine determination of *KRAS*, *NRAS* and *BRAF* mutations in patients with advanced stage of CRC in our institute. For approximately 10% of samples, DNA quality extracted from formalin-fixed paraffin embedded (FFPE) samples does not reach quality criteria and amplicon-based sequencing is not possible or leads to non-interpretable results. In our routine practice, Idylla platform (Biocartis, Mechelen, Belgium) is used as a second-line assay for samples that don’t reach sufficient quality criteria for NGS analysis.

In this study, we describe the integrated routine workflow used in our laboratory based on NGS testing and Idylla automated real-time PCR platform for low quality DNA samples for the determination of *KRAS*, *NRAS* and *BRAF* mutations for patients with mCRC.

## Methods

### Patients and samples

From May 2017 to May 2018, *KRAS*, *NRAS* and *BRAF* mutations have been assessed in a total of 669 FFPE samples (primary tumor or metastases) from patients with mCRC in our Institute. All samples were assessed for *KRAS*, *NRAS* and *BRAF* mutations in the routine management of their cancer. All patients give their consent for the detection of tumor mutations of *KRAS*, *NRAS* and *BRAF* genes. All data were anonymized prior to analysis for this study. This study has been approved by the ethical and scientific board of Institut de Cancérologie de Lorraine.

### Workflow

Tumor specimens have been macrodissected after hematoxylin-eosin slide examination and tumor nuclei content determination by a senior pathologist. DNA has been extracted using QIAamp DNA FFPE tissue kit (Qiagen, Hilden, Germany). After extraction, DNA concentration was measured using the Qubit dsDNA HS assay kit (which allows detection of concentrations between 0.01 and 100 ng/μL) (Qubit 3.0 Fluorometer, ThermoFisher Scientific Inc, Massachusetts, USA). TruSeq FFPE DNA Library Prep QC kit (Illumina, San Diego, USA) and qPCR using Cobas z480 (Roche Diagnostics, Meylan, France) were then used for DNA quality assessment. The PCR commercial kit is qPCR-based able to evaluate DNA fragmentation.

Two nanograms of DNA have been used for qPCR. DNA quality criteria is based on cycles of quantification (Cq) measurement. Cq is defined as the number of cycles needed to reach the threshold set for the assay. Delta-QC (ΔQC) have been calculated using LightCycler 480 Software W UDF 2.0.0 (Roche Diagnostics) by subtracting the Cq values of patient’s samples (= sample to test) from the Cq value of the kit’s internal control (= good quality DNA reference). Cq value will be low or high according to sample DNA quality which can be good or poor, respectively. Thus, a sample with good DNA quality will result in a low ΔQC value and a sample with degraded DNA will result in a high ΔQC value.

**Fig 1:**
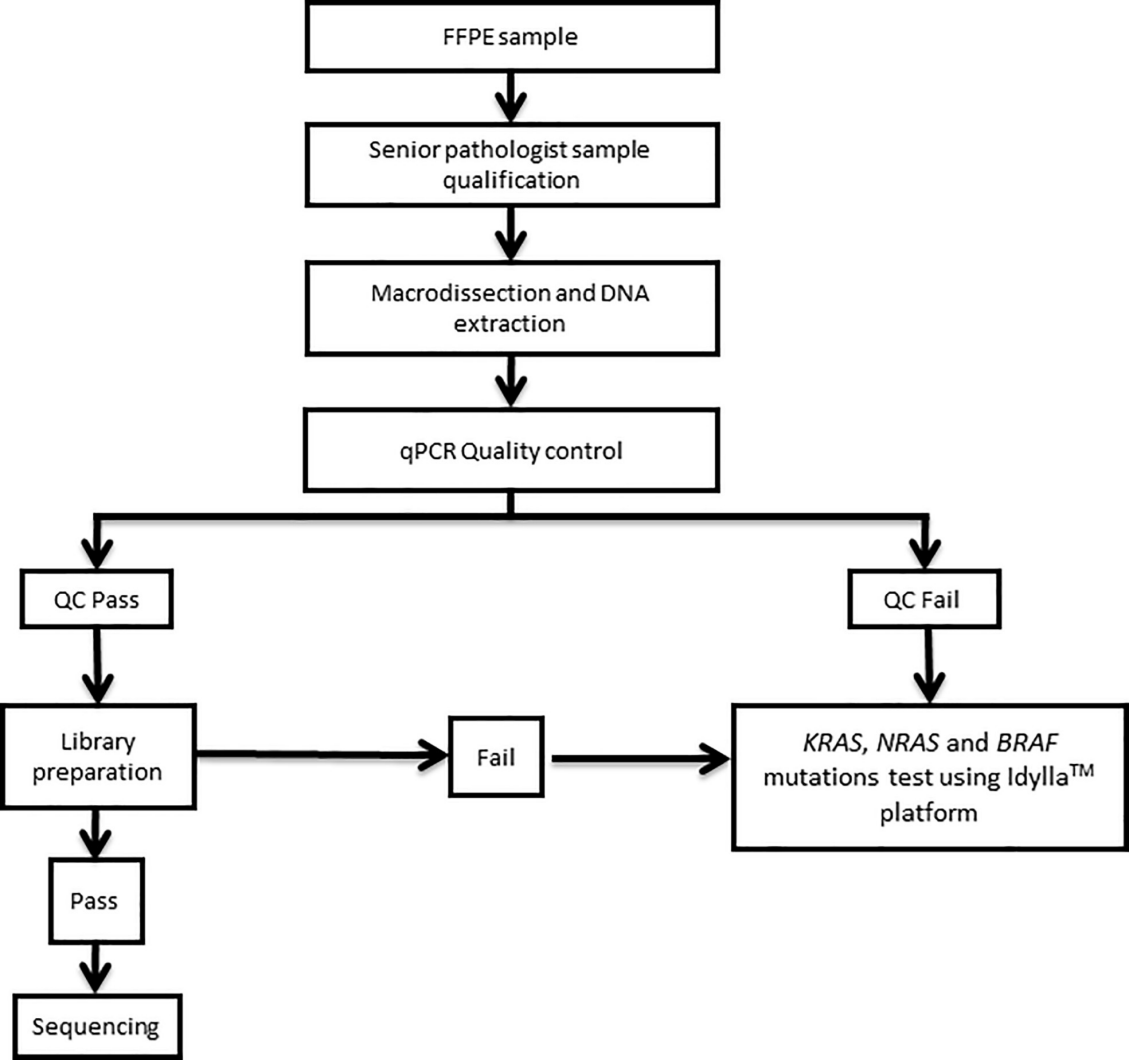
Routine workflow used in our laboratory. DNA quality control is assessed for all samples. Samples that don’t reach quality criteria are assessed using Idylla assay.

According to manufacturer’s protocol, DNA library were then prepared using TruSeq Custom Amplicon Library Preparation Kit v1.5 (Illumina, San Diego, CA, USA) for samples with ΔQC score lower than 6. Idylla platform was used for ΔQC score greater than 6 or for samples that failed at the library preparation step (**Fig 1**).

### Next generation sequencing assay

NGS library was prepared using the “Panel INCa” TruSeq Custom Amplicon Library Preparation Kit v1.5 (Illumina) that includes 16 genes of interest in theranostic, including *KRAS* (full exons 2, 3 and 4), *NRAS* (full exons 2, 3 and 4) and *BRAF* (full exons 11 and 15). The TruSeq Custom Amplicon Library Preparation Kit consists in two separate oligo pools (CATA and CATB). The two oligo pools were hybridized to DNA samples. The specific hybridized targets were ligated, extended and PCR amplified with adapters containing index with specific barcode sequences. Two complementary libraries were generated by targeting the forward and reverse DNA strands. A purification using AMPure XP beads was then performed on the PCR amplified amplicon libraries obtained for non-specific products and reaction components removal.

Libraries DNA concentration were quantified using Qubit 3.0 Fluorometer (ThermoFisher) and their quality was assessed on Fragment Analyzer (Agilent, Advanced Analytical, Ankeny, USA) using Standard Sensitivity NGS Fragment Analysis Kit (Advanced Analytical). PCR products sizes target was 260bp. All libraries were normalized to enable similar amplification and sequencing levels for each sample library within the same run. Sequencing was performed according to the manufacturer’s protocol. All libraries were pooled prior to sequencing on the MiSeq instrument (Illumina). After sequencing, data were treated with Sophia DDM software v.4.3 (Sophia genetics, Saint-Sulpice, Switzerland). Sequences were aligned using reference genome GRCh37/hg19. For patients with mCRC, *KRAS*, *NRAS* and *BRAF* mutational statuses were systematically reported to the clinician as well as relevant mutations in other genes for genomics guided clinical trials.

### Idylla *KRAS* and *NRAS-BRAF* mutation test

The Idylla platform is a fully cartridge-based automated platform which uses microfluidics processing with all reagents on-board [8].

We used two different cartridges for this study. Idylla *KRAS* mutation test detects 21 mutations on the *KRAS* gene (exons 2, 3 and 4) and the Idylla *NRAS-BRAF* mutation test detects 18 mutations on the *NRAS* gene (exons 2, 3 and 4) and 5 on the *BRAF* gene (exon 15). Limit of detection (LOD) depends on each mutation is 1% for *BRAF* gene’s mutations, between 1 and 8.5% for mutations on the *NRAS* gene and between 1 and 5% for mutations on the *KRAS* gene. LOD per mutation are described in S1 and S2 Tables.

Briefly, FFPE tissue section was “sandwiched” in filter papers and introduced in the cartridge according to manufacturer’s protocol. The tissue area of the FFPE specimen should minimally be 50 mm^2^ when 5 μm FFPE tissue sections are used or 25 mm^2^ when 10 μm FFPE tissue sections are used. If tissue area with one section is less than required multiple FFPE tissue sections will be used. All samples in this study have been run in accordance with the manufacturer’s recommendations. After 130 min for Idylla *KRAS* mutation test and 110 min for Idylla *NRAS-BRAF* mutation test, final reports were directly available on the Idylla console. Results were presented as “no mutation detected”, “mutation detected in gene X (*KRAS*, *NRAS* or *BRAF*) codon XX” or “invalid”.

**Fig 2:**
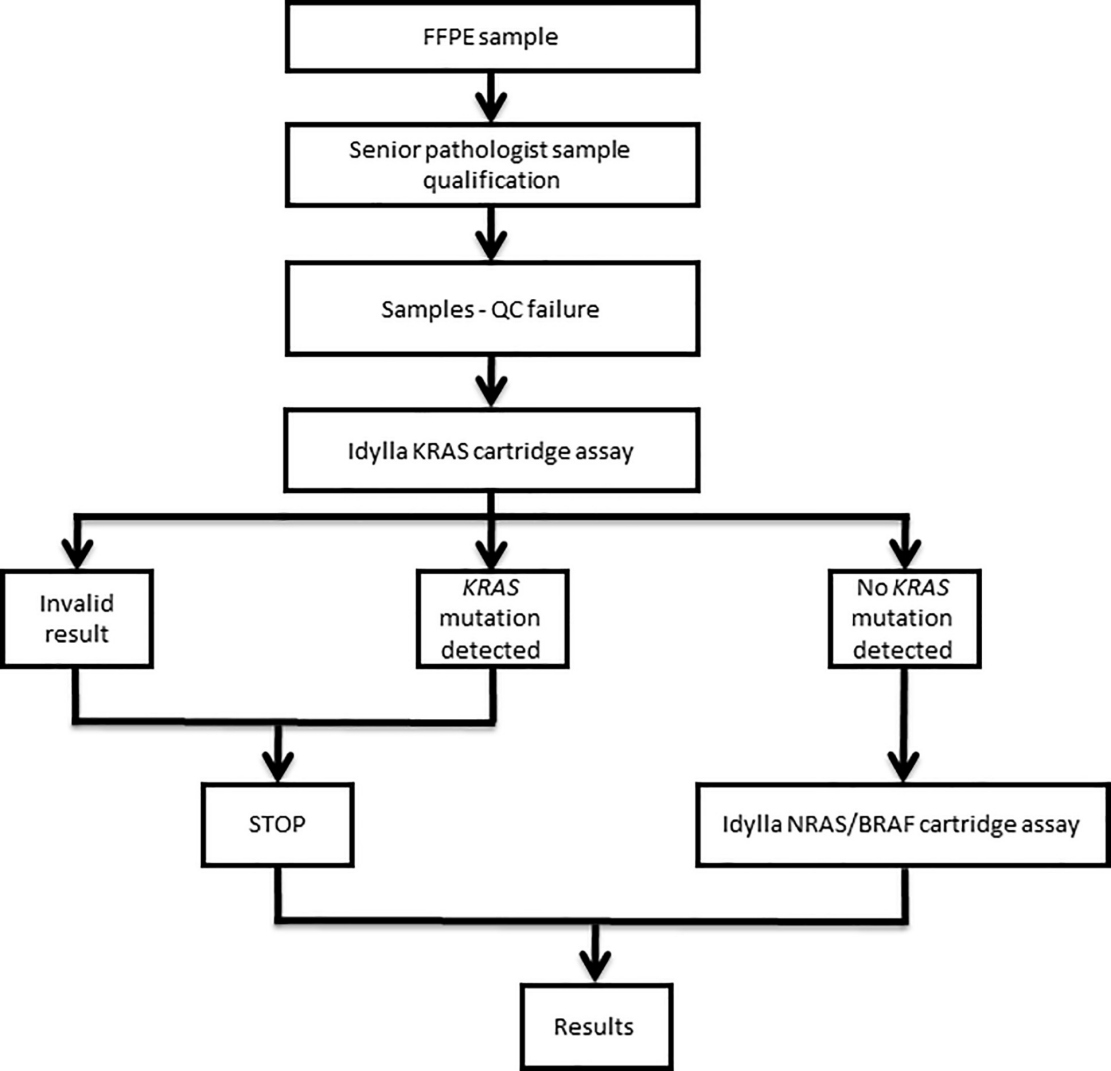
Decision algorithm used in our laboratory for the determination of *KRAS*, *NRAS* and *BRAF* mutations using the Idylla system.

For each poor DNA quality sample, the required number of 5 or 10 μm FFPE tissue section was introduced in a *KRAS* cartridge. For « invalid » or « mutated » results, the process was stopped and no further test was assessed. For samples with no *KRAS* mutation detected, a Idylla *NRAS-BRAF* mutation test was used (**Fig 2**). A “post-analytic” slide corresponding to an extra FFPE slide after the slides introduced in the Idylla cartridges was systematically prepared for hematoxylin-eosin stain and re-evaluation of the tumor nuclei content by a pathologist to ensure the presence of tumor cells in the analyzed samples.

## Results

Among 669 samples analyzed in routine from May 2017 to May 2018, 67 samples failed to reach the quality requirement (ΔQC > 6, n=53) or reached the quality requirement (ΔQC ≤ 6, n=14) but did not allow library preparation or gave invalid results after DNA sequencing.

A total of 67 samples were thus tested using Idylla assay. Among 67 samples, 17 samples had an invalid result with Idylla *KRAS* mutation assay (25.4%) and 50 samples (74.6%) had interpretable results. For 50 patients, samples were successfully recovered using Idylla assay and an interpretable result has been addressed to the oncologist. In total, *KRAS*, *NRAS* or *BRAF* results were available for 652 samples (97.4%).

Among 67 samples, 14 have a ΔQC ≤ 6 (20.9%), 32 have a ΔQC between 6 and 10 (47.8%) and 21 have a ΔQC ≥ 10 (31.3%). Among the 14 samples with ΔQC ≤ 6, 13 samples (92.9%) were recovered by Idylla. Among 32 samples, 30 (93.6%) with a ΔQC between 6 and 10 have been recovered by Idylla. Finally, among the 21 samples which failed NGS with ΔQC > 10, 6 samples (28.6%) have been recovered by Idylla (**Table 1**).

**Table 1:** Samples recovered by Idylla according to ΔQC.

ΔQC	Number of samples	Number of samples recovered with Idylla assay
≤ 6	14	13 (92.9%)
> 6 and < 10	32	30 (93.7%)
≥ 10	21	6 (28.6%)
Total	67	49 (73.1%)

**Fig 3:**
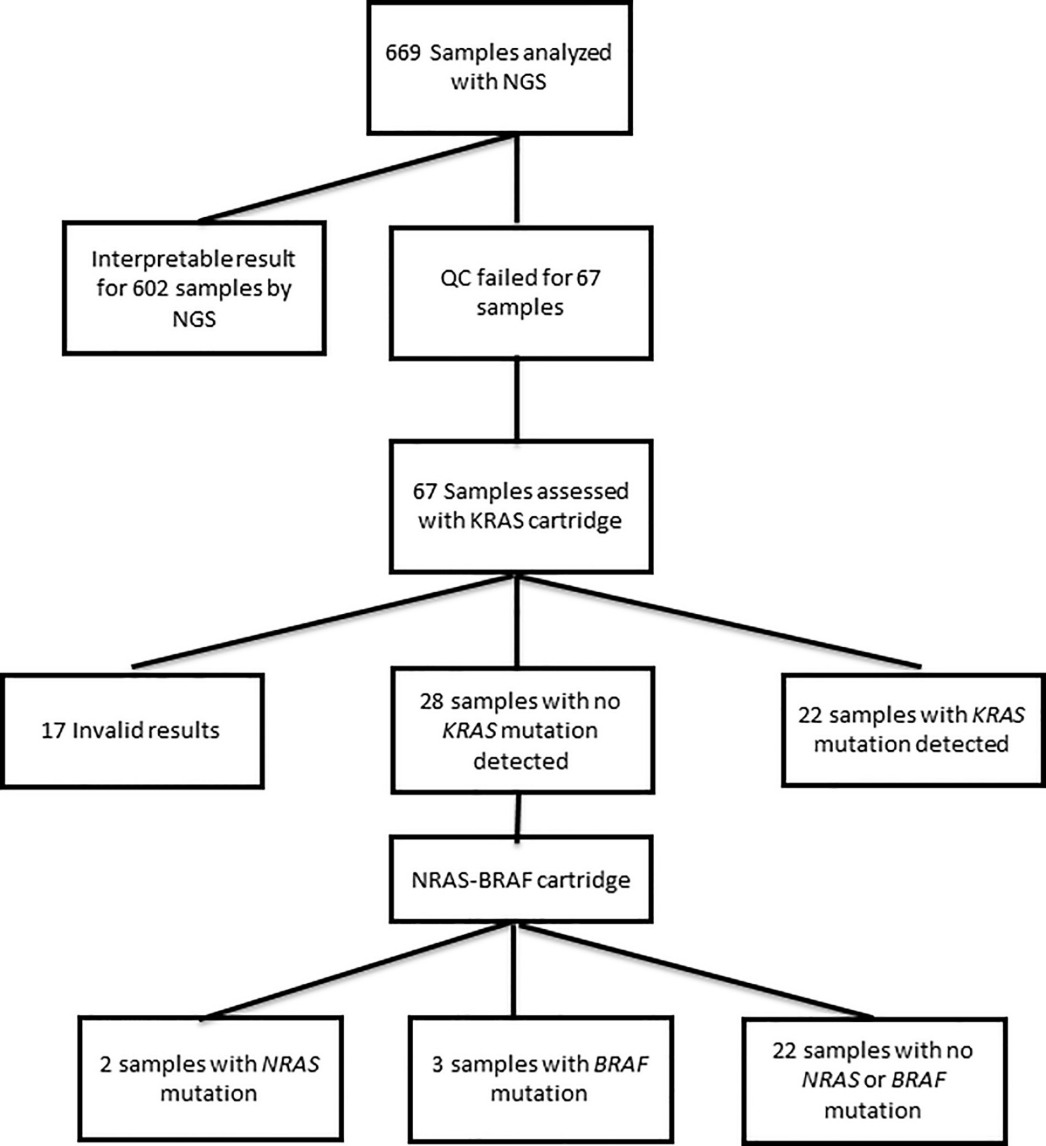
A total of 669 mCRC samples have been analyzed. 53 mCRC samples failed QC for NGS assessment and 14 samples failed a valid sequencing result and have been analyzed with Idylla *KRAS* cartridge. No further analysis was conducted for the 17 invalid results and the 22 samples with a *KRAS* mutation. 28 wild-type *KRAS* samples have been analyzed with Idylla *NRAS-BRAF* mutation test.

Among the 50 samples with an interpretable result with Idylla *KRAS* mutation test, 22 (44%) had a *KRAS* mutation and 28 (56%) were wild-type for *KRAS* **(Table 2)**. According to our standard operating procedure, these 28 *KRAS* wild-type samples have been tested with Idylla *NRAS-BRAF* mutation test. Among 28 samples, 2 samples had a *NRAS* mutation (7.1%), 3 (10.7%) a *BRAF* mutation and 22 (78.6%) samples were wild-type for *NRAS* and *BRAF*. One sample had an invalid result (3.6%). All mutations results are detailed in **Table 3**. Among 67 samples that were not suitable for NGS testing and tested with Idylla assay, 27 (40.3%) had a mutation with a direct clinical impact for the management of mCRC (**Fig 3**).

**Table 2:** *KRAS*, *NRAS* and *BRAF* results using Idylla assay.

Mutation		Number of samples using Idylla testing (%)
***KRAS***	Total	22 (32.8%)
	Codon 12	16
	Codon 13	3
	Codon 61	1
	Codon 117	1
	Codon 146	1
***NRAS***	Total	2 (3.0%)
	Codon 12	1
	Codon 61	1
***BRAF***	Total	3 (4.5%)
	Codon 600	3
**Wild-type**	Total	22 (32.8%)

**Table 3:** Samples results including QC, ΔQC, DNA concentration (ng/μL) and mutation results for *KRAS*, *NRAS* and *BRAF* genes using the Idylla system.

Sample	Percentage tumor cells content	QC	ΔQC	DNA concentration (ng/μl)	Result Idylla *KRAS*	Result Idylla *NRAS*	Result Idylla *BRAF*
**1**	60%	34.9	16.3	7.8	WT	WT	codon 600 mutation
**2**	40%	35.2	16.6	6.9	WT	codon 12 mutation	WT
**3**	30%	23.2	4.5	0.7	WT	WT	WT
**4**	20%	31.8	13.6	4.3	Invalid	Not tested	Not tested
**5**	40%	26.2	5.9	0.9	codon 117 mutation	WT	WT
**6**	20%	23.5	5.3	6.3	codon 12 mutation	Not tested	Not tested
**7**	50%	29.8	9.5	3.4	WT	WT	WT
**8**	50%	29.3	9.1	1.2	WT	Invalid	Invalid
**9**	60%	27.4	8.1	25	WT	WT	WT
**10**	60%	27	7.6	0.9	codon 12 mutation	WT	WT
**11**	80%	34	16.3	0.1	Invalid	Not tested	Not tested
**12**	20%	26	8.9	17.8	codon 12 mutation	WT	WT
**13**	60%	26.4	8.7	5.9	codon 12 mutation	Not tested	Not tested
**14**	25%	34.1	16.4	0.7	Invalid	Not tested	Not tested
**15**	40%	28.1	10.9	0.3	Invalid	Not tested	Not tested
**16**	40%	24.9	7.2	6.3	codon 12 mutation	Not tested	Not tested
**17**	60%	33.3	15.7	0.6	Invalid	Not tested	Not tested
**18**	40%	27.4	9.8	7.8	WT	WT	WT
**19**	70%	20.9	3.7	1	WT	WT	WT
**20**	80%	21.2	4	0.8	codon 61 mutation	Not tested	Not tested
**21**	30%	30.5	11.7	2.5	WT	WT	WT
**22**	70%	20.4	2.8	3.1	codon 12 mutation	Not tested	Not tested
**23**	40%	32.1	13.3	0.1	Invalid	Not tested	Not tested
**24**	40%	23.3	6.1	11.8	WT	WT	WT
**25**	40%	21.4	2.6	2.7	WT	WT	WT
**26**	30%	24.6	5.7	1.7	WT	WT	WT
**27**	80%	19.3	1.7	0.4	WT	WT	WT
**28**	50%	22.8	5.1	7.8	WT	WT	WT
**29**	40%	30.1	12.4	0.4	Invalid	Not tested	Not tested
**30**	70%	27.2	9.5	12.9	WT	codon 61 mutation	WT
**31**	70%	23.3	6	14.6	codon 12 mutation	Not tested	Not tested
**32**	30%	26.1	8.8	out of range	Invalid	Not tested	Not tested
**33**	10%	23.9	7	29.8	codon 12 mutation	Not tested	Not tested
**34**	20%	24.7	6.9	13	WT	WT	codon 600 mutation
**35**	85%	21.6	4.7	3.9	WT	WT	WT
**36**	60%	24.9	7.1	16.1	WT	WT	codon 600 mutation
**37**	60%	24.4	6.1	11.5	codon 12 mutation	Not tested	Not tested
**38**	60%	30.7	12.4	6.2	WT	WT	WT
**39**	50%	24.6	6.8	2	WT	WT	WT
**40**	70%	28.2	6.5	22	codon 12 mutation	Not tested	Not tested
**41**	70%	33.6	11.9	1.3	Invalid	Not tested	Not tested
**42**	70%	37.1	18.9	2.9	Invalid	Not tested	Not tested
**43**	60%	33.1	13.8	8	Invalid	Not tested	Not tested
**44**	5%	28.8	11.3	2.4	Invalid	Not tested	Not tested
**45**	70%	25.5	6.3	35.7	codon 12 mutation	Not tested	Not tested
**46**	10%	26.4	7	11.1	WT	WT	WT
**47**	80%	26.7	6.6	27.7	WT	WT	WT
**48**	< 10%	25.4	5.3	7.3	Invalid	Not tested	Not tested
**49**	15%	25.6	6.3	12.3	codon 12 mutation	Not tested	Not tested
**50**	30%	28.4	9.9	1.9	codon 12 mutation	Not tested	Not tested
**51**	15%	25.8	6.9	21.9	WT	WT	WT
**52**	80%	25.9	7	7.5	codon 13 mutation	Not tested	Not tested
**53**	50%	31	13.3	7.1	Invalid	Not tested	Not tested
**54**	90%	23.6	6.2	21.3	WT	WT	WT
**55**	20%	24.9	7.4	0.6	WT	WT	WT
**56**	10%	26.2	8.7	44.3	WT	WT	WT
**57**	30%	34.4	15.5	1.4	Invalid	Not tested	Not tested
**58**	40%	27.7	8.9	3.5	codon 13 mutation	Not tested	Not tested
**59**	30%	25.6	8	64.7	codon 146 mutation	Not tested	Not tested
**60**	50%	29.7	12.1	46.2	codon 13 mutation	Not tested	Not tested
**61**	70%	31.5	14	14.7	codon 12 mutation	Not tested	Not tested
**62**	20%	25.3	7.9	10.5	codon 12 mutation	Not tested	Not tested
**63**	70%	23.5	6.1	44.9	codon 12 mutation	Not tested	Not tested
**64**	30%	27.6	10.2	1.1	Invalid	Not tested	Not tested
**65**	50%	20.6	4	5.4	WT	WT	WT
**66**	20%	29.3	12.7	2.8	Invalid	Not tested	Not tested
**67**	15%	25.4	7.1	25.4	WT	WT	WT

Among the 602 samples with valid results with NGS, 12 (2%) had an uncommon mutational profile with non-hotspot mutations uncovered by the Idylla platform.

## Discussion

In this study, we retrospectively collected data from 669 samples from patients with mCRC for *KRAS*, *NRAS* and *BRAF* mutational status assessment. DNA was of too poor quality for a NGS analysis for 10% of samples. The most common cause of DNA degradation in FFPE tissues is an inappropriate length of tissue fixation. In the large majority of the cases, the same poor DNA quality is found in a second block from the same lesion, because of the pre-analytical steps were similar. Moreover, asking a second block to the pathologist can improve delays for *RAS* and *BRAF* testing. In our experience, analyzing poor quality DNA (ΔQC > 6) using amplicon-based NGS assay leads to no library amplification or to results with a very high background noise which is not compatible with a good interpretation for samples with variant allele frequency (VAF) under 10% or more. The risk of false positive or false negative is too high when sequencing poor quality DNA, thus we chose a second assay which is less stringent on DNA quality to avoid a second biopsy to the patient. These samples have been analyzed with the Idylla platform and 75% of the samples have been retrieved. We assume that Idylla assay amplifies shorter amplicons than the library preparation kit we use and is then less influenced by DNA fragmentation than NGS assay. We tested this hypothesis by analyzing DNA fragments sizes from samples with a range of ΔQC and the results confirmed that a high ΔQC is associated with shorter DNA fragments (S1 Fig). Fourteen samples were found with a ΔQC ≤ 6 but did not allow library preparation or gave non interpretable results after sequencing. We assume that for these samples, ΔQC close to 6 combined with low DNA yield led to a library preparation failure and or high background noise. Using shorter amplicons for library preparation or using capture-based sequencing may address this issue.

Sample #8 had a valid result (WT) with *KRAS* cartridge and invalid result with *NRAS-BRAF* cartridge. The same quantity of FFPE sections have been used in the two cartridges, which suggests that DNA quality requirements may depends on the type of cartridge or a high DNA fragmentation in *NRAS* and *BRAF loci*.

Two strategies are commonly used for tumor driver mutations assessment: broad approach as NGS and targeted approach as PCR-based methods. NGS allows the exhaustive DNA analysis of regions of interest with no or few limitations. The targeted approach is only based on the detection of hotspot mutations, thus can’t detect rare mutations. Among the analyzed samples, 2% were found with a non-hotspot mutation on *KRAS*, *NRAS* or *BRAF* genes with NGS. By its design and its targeted approach the Idylla assay can’t detect these mutations. Whereas only mutations on codons 12, 13, 59, 61, 117 and 146 are validated resistance mutations to anti-EGFR mAbs, we have shown that non-hotspot *KRAS* or *NRAS* mutations may have an impact on resistance to anti-EGFR mAbs [9–11]. In the absence of more published data on rare mutations and resistance to anti-EGFR mAbs, targeted detection of *KRAS*, *NRAS* and *BRAF* mutation remains relevant and is recognized as the golden standard. In our experience, most of the sequencing prescriptions for patients with mCRC are used to choose the first-line therapy, thus use of NGS is only useful in a molecular tumoral board context.

DNA quality can sometimes make NGS analysis not possible; this assay also requires a minimal quantity of DNA to avoid false negative results. DNA quantity can sometimes be an issue with small tissue biopsies. Targeted assays often require less DNA than NGS and are feasible with DNA of poor quality. However, the development of capture-based NGS assays makes sequencing of DNA possible with few quantities of DNA and lower DNA quality [12]. However the Idylla system also has limitations since this assay requires a minimal amount of FFPE material (50 mm^2^ with 5μm tissue section and 25 mm^2^ with 10 μm tissue sections) with a tumor cell percentage superior to 10%.

NGS is known as an expensive assay and requires a highly-qualified team [13]. The analysis of raw sequencing data is highly-dependent upon bioinformatics and all detected variants need to be analyzed and biologically interpreted in the clinical context. These several steps mostly take from 3 to 5 days from DNA extraction to interpretation of the data. The targeted PCR assay we chose here is really easy to use and requires less than 5 minutes hands-on time. The results are directly available at the end of the analysis which requires 110 to 130 minutes depending on the test [8]. Idylla testing has been evaluated in several studies which have shown an excellent concordance with reference methods [14,15].

In our study, 97.3% of samples had an interpretable result using our integrated routine workflow. Avoiding a new biopsy which could be no possible nor ethical, we assume that this workflow allows to commence first-line therapy earlier for patients with mCRC.

We chose in our routine workflow to use a broad approach first and a targeted approach as a second choice. In some theranostics indications, it may sound relevant to firstly use a targeted approach and use NGS only for tumor molecular board purposes.

Indeed, turnaround time for gene panel analysis from sample reception to final results is in average 3 to 4 days whereas final results with Idylla system require only one day. Moreover, to optimize time and sequencing costs, most of routine laboratories use samples batches implying longer delays.

According to the French healthcare system, a targeted analysis of *RAS* and *BRAF* is evaluated to 556.20 euros (440.10 € for the analysis of the 6 exons of *KRAS* and *NRAS* and 116.10 € for the research of the V600 mutations of *BRAF*), whereas a gene panel with a size inferior to 20Kb is evaluated to 882.90 €.

This study focuses on the management of patients with mCRC, but *KRAS*, *NRAS* or *BRAF* mutations testing is also relevant in other cancers like metastatic melanoma or non-small cell lung cancer [16,17].

The Idylla system has been built to work with FFPE slides directly in the cartridge, but it has recently been showed that the detection of *EGFR* mutations was possible by adding DNA directly in the cartridge which can also be an advantage since DNA already extracted for the NGS assay can also be tested using Idylla without using more fixed-tissue [17,18]. For patients who require commencing a targeted therapy quickly, a two steps scheme using extracted DNA in Idylla first and then NGS, may be used, saving time and tissue.

In conclusion, our study shows that an integrated workflow that uses NGS and Idylla platform allows the determination of mutational status of *KRAS*, *NRAS* and *BRAF* genes of 97.4% of samples and can retrieve 75% of samples that don’t reach quality criteria for NGS testing.

## Supporting information

S1 Fig DNA Fragmentation profiles (Fragment analyzer) for samples with a range of ΔQC between 0.1 and 15. The size of DNA fragments decreases when ΔQC increases.
(PDF)

S1 Table Limit of detection for the different mutations covered by the Idylla KRAS mutation test. (DOCX)

S2 Table Limit of detection for the different mutations covered by the Idylla NRAS-BRAF mutation test. (DOCX)
