# Supplementary material for: Integrated routine workflow using next-generation sequencing and a fully-automated platform for the detection of KRAS, NRAS and BRAF mutations in formalin-fixed paraffin embedded samples with poor DNA quality in patients with colorectal carcinoma
Source: PLoS One. 2019 Feb 27;14(2):e0212801. doi: 10.1371/journal.pone.0212801 (PMC6392303; doi:10.1371/journal.pone.0212801)
Supplement: S1 Fig — The size of DNA fragments decreases when ΔQC increases. (PDF) [file pone.0212801.s001.pdf]

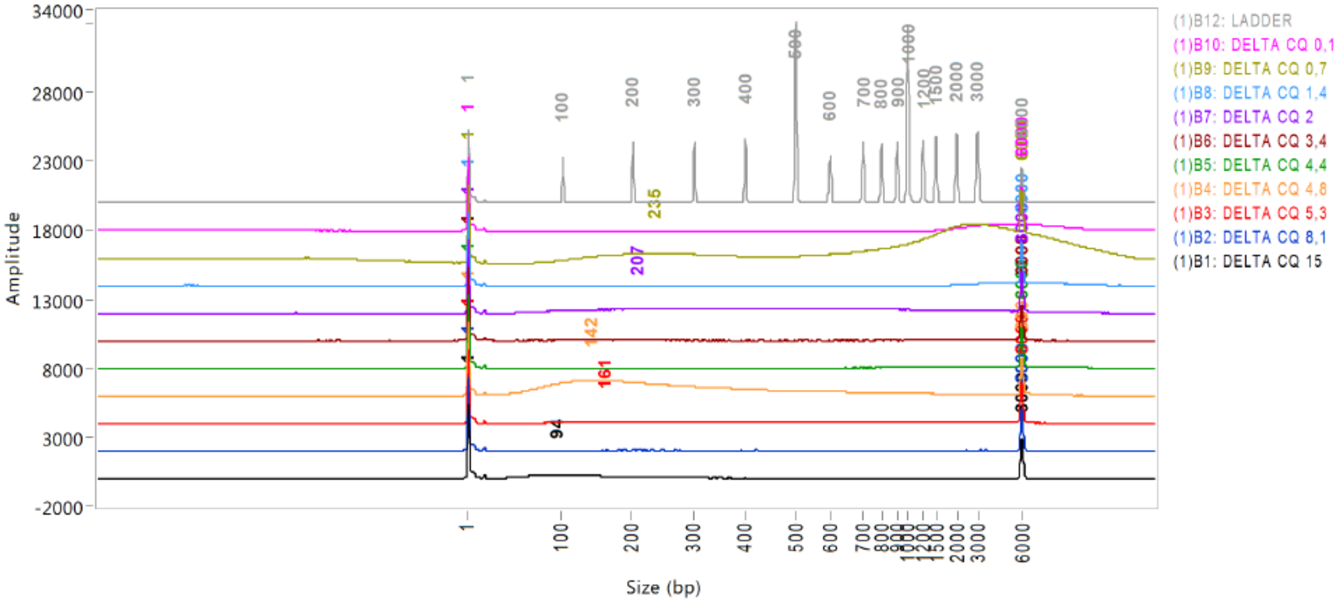

Figure S1: DNA Fragmentation profiles (Fragment analyzer) for samples with a range of ΔQC between 0.1 and 15. The size of DNA fragments decreases when ΔQC increases.
