# Supplementary material for: Integrated routine workflow using next-generation sequencing and a fully-automated platform for the detection of KRAS, NRAS and BRAF mutations in formalin-fixed paraffin embedded samples with poor DNA quality in patients with colorectal carcinoma
Source: PLoS One. 2019 Feb 27;14(2):e0212801. doi: 10.1371/journal.pone.0212801 (PMC6392303; doi:10.1371/journal.pone.0212801)
Supplement: S1 Table — (DOCX) [file pone.0212801.s002.docx]

**S1 table:** limit of detection for the different mutations covered by the Idylla^TM^ KRAS mutation test

| **Amino acid change** | **Coding DNA change** | **Limit of detection** |
| --- | --- | --- |
| p.(Gly12Ala) | c.35G>C | 5.0% |
| p.(Gly12Cys) | c.34G>T | 5.0% |
| p.(Gly12Asp) | c.35G>A | 5.0% |
| p.(Gly12Arg) | c.34G>C | 1.0% |
| p.(Gly12Ser) | c.34G>A | 5.0% |
| p.(Gly12Val) | c.35G>T | 5.0% |
| p.(Gly13Asp) | c.38G>A | 5.0% |
| p.(Ala59Glu) | c.176C>A | 5.0% |
| p.(Gln61His) | c.183A>C | 5.0% |
| p.(Gln61Lys) | c.181C>A; c.180_181delinsAA | 5.0% |
| p.(Gln61Leu) | c.182A>T | 5.0% |
| p.(Lys117Asn) | c.351A>C; c.351A>T | 5.0% |
| p.(Ala146Thr) | c.436G>C | 5.0% |

**KRAS**

**MATERIAL**

**ALLELIC**
