# Supplementary material for: Integrated routine workflow using next-generation sequencing and a fully-automated platform for the detection of KRAS, NRAS and BRAF mutations in formalin-fixed paraffin embedded samples with poor DNA quality in patients with colorectal carcinoma
Source: PLoS One. 2019 Feb 27;14(2):e0212801. doi: 10.1371/journal.pone.0212801 (PMC6392303; doi:10.1371/journal.pone.0212801)
Supplement: S2 Table — (DOCX) [file pone.0212801.s003.docx]

**S2 table:** limit of detection for the different mutations covered by the Idylla^TM^ NRAS-BRAF mutation test

|  | **Amino acid change** | **Coding DNA change** | **Limit of detection** |
| --- | --- | --- | --- |
| *NRAS* | p.(Gly12Asp) | c.35G>A | 1.0% |
|  | p.(Gly12Cys) | c.34G>T | 1.0% |
|  | p.(Gly12Ser) | c.34G>A | 5.5% |
|  | p.(Gly12Val) | c.35G>C | 1.0% |
|  | p.(Gly12Ala) | c.35G>T | 1.0% |
|  | p.(Gly13Asp) | c.38G>A | 1.5% |
|  | p.(Gly13Arg) | c.37G>C | 1.0% |
|  | p.(Gly13Val) | c.38G>A | 1.0% |
|  | p.(Ala59Thr) | c.175C>A | 3.0% |
|  | p.(Gln61His) | c.183A>C; c.351G>T | 1.0% |
|  | p.(Gln61Lys) | c.181C>A | 1.3% |
|  | p.(Gln61Leu) | c.182A>T | 1.3% |
|  | p.(Gln61Arg) | c.182A>G | 1.6% |
|  | p.(Lys117Asn) | c.351A>C; c.351A>T | 1.0% |
|  | p.(Ala146Thr) | c.436G>C | 8.5% |
|  | p.(Ala146Val) | c.437C>T | 1.0% |
| *BRAF* | p.(Val600Glu) | c.1799T>A; c.1799_1800delinsAA ; c.1799_1800delinsAC ;  c.1798_1799delinsAA ;  c.1798_1799delinsAG | 1.0% |
